# Increase in the Number of Tuberculosis Cases Treated following Tuberculin Skin Testing in First-Year Schoolchildren in Madagascar

**DOI:** 10.1371/journal.pone.0095494

**Published:** 2014-04-17

**Authors:** Rila Ratovoson, Voamalala Raharimanga, Niaina Rakotosamimanana, B. Ravaloson, Maherisosa Ratsitorahina, Rindra Randremanana, Herimanana Ramarokoto, Soatiana Rajatonirina, Voahangy Rasolofo, Vincent Richard

**Affiliations:** 1 Epidemiology Unit, Pasteur Institute in Antananarivo, Antananarivo, Madagascar; 2 Mycobacteria Unit, Pasteur Institute in Antananarivo, Antananarivo, Madagascar; 3 Tuberculosis Treatment Center, Ministry of Health, Antananarivo, Madagascar; 4 Epidemiology Unit, Pasteur Institute in Dakar, Dakar, Senegal; Glaxo Smith Kline, Denmark

## Abstract

**Background:**

Tuberculosis continues to cause unacceptably high levels of disease and death worldwide. Active preventive strategies are required to improve tuberculosis control and to increase the number of cases treated in developing countries. The aim of this study was to evaluate the utility of the tuberculin skin test (TST) in first-year schoolchildren as a means of increasing the number of tuberculosis cases detected through the screening of close contacts.

**Methods:**

All members of the households of 90 schoolchildren assigned to three groups on the basis of TST category (≤5 mm, [5–15)mm, ≥15 mm) were screened for sputum smear-positive pulmonary tuberculosis. The percentage detection of tuberculosis in close contacts was compared between TST categories.

**Results:**

We identified 433 close contacts of the 90 schoolchildren, who were then evaluated for tuberculosis. We identified 11 cases of pulmonary tuberculosis among the close contacts (7 already on treatment and 4 previously undiagnosed): 0 in TST category ≤5 mm, 3 in TST category [5–15) mm and 8 in TST category ≥15 mm). This approach increased the detection of tuberculosis cases by a factor of 1.6 in first-year schoolchildren of the TST ≥5 mm group.

**Conclusion:**

TST in first-year schoolchildren is a potentially effective method for improving the detection of tuberculosis in close contacts.

## Introduction

Tuberculosis (TB) continues to be a major public health problem worldwide. According to the World Health Organization (WHO), 8.6 million people presented with symptoms of tuberculosis (usually a productive cough and fever) in 2012 and 1.3 million people, 95% of whom were from from low- and middle-income countries, died from tuberculosis [1]. Tuberculosis is a particularly serious public health problem in developing countries, which account for 95% of tuberculosis cases and 98% of tuberculosis deaths [2]. In Madagascar, 18,000 to 20,000 new cases of tuberculosis are detected each year [3], [4]. The incidence of TB in Madagascar is high, the most recently reported value being 266 cases per 100,000 inhabitants in 2010, according to a World Bank report released in 2011 [5]. In the last few years, the WHO has recommended that countries implement several interventions to help control the spread of tuberculosis, through measures improving the prevention, diagnosis and treatment of the disease. One goal for TB control is the detection of 84% of new, smear-positive TB cases and the curing of 87% of these cases by 2015 [6]. This will require improvements in case identification, which will in turn require the identification of sources of infection in a community. It has been suggested that previously unrecognized cases of TB could be detected by investigating the close contacts of children with significant tuberculin reactions [7]. Following the discovery of TB in children, associated investigations can result in the discovery of undiagnosed adult smear-positive cases [7]. However, in countries in which the Bacillus Calmette-Guerin vaccination is administered at birth, TST interpretation in children may be complicated by this prior vaccination. However, some studies [8], [9] have shown that TST reactivity gradually declines over the first seven years of life, paralleling the decline in neonatal BCG-induced reactions. One recent study in Madagascar showed that BCG vaccination status had no evident effect on TST reactivity in first-year schoolchildren in Antananarivo. [5]. As younger children are presumed to have more limited exposure to individuals outside their own homes, the most likely source of infection in children with positive TST results, particularly for those under the age of six years, is a close contact at home. We tested this hypothesis to assess the relevance of a screening strategy targeting first-year schoolchildren.

The aim of this study was to evaluate a TST-based screening strategy targeting first-year schoolchildren in Madagascar as an active tool for identifying close contacts with sputum smear-positive pulmonary tuberculosis.

## Methods

### Design

This study was carried out in the fourth district of Antananarivo, the capital city of Madagascar. This district was identified by Randremanana *et al*. as having a high incidence of TB [3], [4]. The target population was schoolchildren in the first year of primary school, generally aged between six and eight years. About 60% of the children living in this low-income district attend school. An initial screening was carried out during the systematic medical visits of these children [5], to determine the TST response of each child in a public school. In total, 376 schoolchildren were screened and analyses of the results obtained indicated that prior BCG vaccination had no effect on the TST response of children over the age of six years [5]: 298 (79%) children had a negative TST result, nine had (3%) a 5–10 mm response, 26 (7%) had a 10–15 mm response and 43 (11%) had a response of more than 15 mm.

### Study Population

From the original cohort of schoolchildren, we targeted first-year schoolchildren under the age of eight years with a BCG scar and close contacts of their families. As very few children had TST responses of [5–10) mm or [10–15) mm, we defined only three groups for TST response (<5 mm, [5–15) mm, ≥15 mm). For each group, after informed written consent for participation in the study was obtained from all family members we randomly selected 30 families of BCG-vaccinated schoolchildren. If one or more members of the family refused to participate in the study, the family was not included.

This study was carried out on the various families from April to June 2010. Each family was visited at home by the epidemiology team from the Pasteur Institute of Madagascar.

All family contacts, defined as household members in sustained close contact with the child (living in the same household and/or sharing at least one meal per day with the child) were included. The families were provided with information about the study, and individual consent for participation was then obtained from all family members. Each individual was identified by a study identification number and screened by the same procedures regardless of the TST status of the child concerned.

A standardized procedure was used for each participant (schoolchildren and family members), including standardized questionnaire (age, sex, past or present treatment for TB, reported BCG vaccination), anthropometric assessment (weight and height measurements) and a tuberculin skin test (TST). The TST was administered on the ventral surface of the left forearm, by the intradermal technique, until a weal of 6–10 mm in diameter was visible at the injection site. Participants were provided with information about care of the test region and about the need to read the test reaction 72 hours after the test. Test results were read by epidemiologists trained in the administration/reading of the tuberculin reaction. If the induration induced by the TST was at least 15 mm in diameter, in line with national policy, or if characteristic symptoms of pulmonary tuberculosis were found, a chest X ray and a medical evaluation for TB were carried out by physicians at the national center for tuberculosis diagnosis and treatment. The diagnosis of pulmonary tuberculosis was confirmed by sputum examination, performed by the national center for tuberculosis diagnosis and treatment, which was also responsible for all decisions concerning the treatment of suspected or confirmed cases in this study.

### Ethical Considerations

The protocol was approved by the Malagasy National Ethics Committee. This study was authorized by the Malagasy National Tuberculosis Program (NTP), local health services (CRD, SSD, CSB II), local school services (CISCO) and municipal authorities (CUA, Police, Fokontany). The parent-teacher associations of the schools were consulted and informed about the purpose of the study and the way in which it would be carried out before recruitment and enrolment began. Written permission for screening in the school was obtained from these bodies.

For family contacts, the study was explained to all the members of the family and written informed consent was obtained from all subjects or their guardians, for each member of the family under 18 years of age. If any of the family members refused to participate, the family was not included in the study. The refusal rate was low (less than 5%). The principal benefit to the participants in this study (the schoolchildren and their families) was the screening for TB. If the disease was detected, diagnosis and treatment were free.

### Data Management and Statistical Analysis

An anonymized database was created with Access. A double-entry system was used for data input and we checked for data entry errors. Analyses were conducted with R software [10].

We carried out a descriptive analysis, in which proportions and distributions were determined for each variable category. Fisher’s exact tests were carried out for comparisons of qualitative data and Kruskal-Wallis tests were carried out for comparisons of quantitative data. *P* values <0.05 were considered statistically significant.

## Results

### Analysis of the TST Status of First-year Schoolchildren

The characteristics of the 90 schoolchildren tested are presented as a function of TST result group in table 1. No significant difference in sex or age distribution was found between the three TST groups. The proportion of children with a family contact suffering from TB was higher in the groups with TST responses of at least 5 mm in diameter, at 13% for the [5,15) mm group and 20% for group≥15 mm group. No significant difference was found between the three groups in terms of contact with TB cases outside the household (*p* = 0.07).

**Table 1:** Characteristics of the groups of schoolchildren defined on the basis of TST results.

Characteristics	[0–5) mm *N* = 30	[5–15) mm *N* = 30	≥15 mm *N* = 30	*p*-value
	*n* (%)	*n* (%)	*n* (%)	
Sex				
Male	19 (63)	22 (73)	20 (67)	0.78
Female	11 (37)	8 (27)	10 (33)	
Age				
Mean [95% CI]	6.70 [6.59–6.91]	6.76 [6.61–7.03]	6.80 [6.66–7.07]	0.82
Past BCG vaccination				
with vaccination card	4 (13)	6 (20)	2 (7)	0.37
without vaccination card	26 (87)	24 (80)	28 (93)	
History of TB				
No	30 (100)	30 (100)	30 (100)	–
Household contact with TB				
No	30 (100)	26 (87)	24 (80)	0.03
Yes :	0 (0)	4 (13)	6 (20)	
Pulmonary TB	0	2	5	
Other	0	2	1	
Contact with TB				
No	28 (93)	25 (83)	21 (70)	0.07
Yes	2 (7)	5 (17)	9 (30)	
Pulmonary TB	0	2	5	
Other	2	3	4	

### Analysis of Family Close Contacts by TST Group of the Child

All close contacts of the 90 families (30 in each group) included were investigated, resulting in the inclusion of 433 people in this study. The median number of close contacts per child was five (range: (2–9), mean = 4.9, 95% CI:[4.5–5.2]) and did not differ significantly (*p*-value = 0.72) between the three groups defined on the basis of the TST results for the schoolchildren (Table 2).

**Table 2:** Characteristics of close contacts according to the TST status of the schoolchildren.

	TST result groups for the schoolchildren			
Characteristics	[0–5) mm *n* (%)	[5–15) mm *n* (%)	≥15 mm *n* (%)	*p*-value
Number of close contacts screened	140 (32)	144 (33)	149 (34)	
Median number of family members	4	4	5	
Range	(2–6)	(2–6)	(2–6)	0.72
Sex				
Male	64 (46)	65 (45)	71 (48)	0.91
Female	76 (54)	79 (55)	78 (52)	
Age				
Mean [95% CI]	19.1 [16.5–21.6]	20.4 [17.9–22.9]	20.2 [17.6–22.8]	0.53
Median	13	17	15	0.66
Range	(0–60)	(0–61)	(0–62)	
BCG vaccination				
No	5 (3)	13 (9)	5 (3)	0.06
Yes (without vaccination card)	110 (79)	120 (83)	125 (84)	
Yes (with vaccination card)	12 (9)	2 (1)	9 (6)	
Unknown	13 (9)	9 (6)	10 (7)	
Past TB treatment	1 (1)	4 (3)	3 (2)	0.49
Known contact with TB outside household				
No	108 (77)	92 (64)	94 (63)	0.03
Yes	26 (19)	41 (28)	46 (31)	
Unknown	6 (4)	11 (8)	9 (6)	
TST results				
[0–5) mm	78 (56)	63 (44)	54 (37)	<0.01
[5–15) mm	19 (14)	15 (10)	24 (16)	
≥15 mm	43 (30)	66 (46)	70 (47)	
Symptoms of fever	10 (7)	21 (15)	13 (9)	0.10
Symptoms of cough	22 (16)	34 (24)	26 (17)	0.21
Symptoms of night sweats	26 (19)	30 (21)	19 (13)	0.16
Symptoms of weight loss	15 (9)	15 (10)	34 (22)	<0.01
Chest X Ray				
Carried out	47 (34)	67 (47)	75 (51)	<0.01
TB diagnosis				
New extrapulmonary cases	0 (0)	2 (1)	3 (2)	0.38
New pulmonary cases	0 (0)	1 (1)	3 (2)	0.33
All new cases detected	0 (0)	3 (2)	6 (4)	0.04
Previously identified pulmonary Cases on treatment	0 (0)	2 (1)	5 (3)	0.07
All active pulmonary TB cases	0 (0)	3 (2)	8 (5)	<0.01
All active cases	0 (0)	5 (3)	11 (7)	<0.01

The sex ratio (male/female) was 0.85, with 200 male contacts (46%), and did not differ significantly (*p*-value = 0.91) between the groups (Table 2).

The characteristics of TST responses differed significantly between groups, resulting in a larger number of chest X rays being carried out on the close contacts of schoolchildren with TST responses of more than 5 mm in diameter: 47% for contacts of the TST [5,15) mm group and 51% for the TST ≥15 mm group (Table 2).

Overall, 189 (44%) subjects underwent chest X rays, either because they had symptoms characteristic of TB or because they had a TST response of at least 15 mm in diameter (Table 2). In total, 41 of these subjects were referred to and attended the TB clinic for medical evaluation.

Nine contacts with active disease were identified in this study. For four of these newly identified subjects, sputum smears were positive, confirming the diagnosis of pulmonary TB. The other five subjects had been treated for suspected extrapulmonary disease.

Seven other close contacts were diagnosed with pulmonary TB before the study and were already on treatment (Tables 1 & 2).

TB case identification rates (expressed as a percentage) differed significantly (*p* = 0.04) between the TST-negative group and the other two groups (Table 2).

The screening of close contacts of first-year schoolchildren with TST responses of more than 5 mm in diameter increased the number of pulmonary tuberculosis treated cases by a factor of 1.58 (number of cases/number of known cases: 11/7), and the screening of contacts of children with a TST response of more than 15 mm in diameter increased the number of TB cases identified by a factor of 1.60 (8/5) (Table 2).

## Discussion

In Madagascar, the screening of first-year schoolchildren with the TST could be used as a means of identifying the source of TB among the child’s close contacts. This approach increased the number of pulmonary TB cases treated by up to 60%. These results suggest that this strategy is an appropriate way to address the WHO target for 2015 of increasing the percentage of pulmonary tuberculosis cases treated [6]. The close contacts of pulmonary TB patients are at high risk of TB infection and disease. Most infected individuals are asymptomatic and non-infectious. Several whole-blood assays have been developed in the last few years, but the tuberculin skin test (TST) remains the least expensive method available for the screening of *M. tuberculosis* infection in developing countries, despite its well known limitations in terms of accuracy and reliability. In India, a country with a high TB burden, the accuracy of the TST has been shown to be similar to that of Quantiferon [11].

The rationale behind investigations of the close contacts of children in their first year at primary school is that young children with a positive TST result must, by definition, have been recently infected. Younger children are also presumed to have more limited exposure to individuals with pulmonary TB disease outside their own homes, making the identification of a source case among household members more likely than for older subjects [7]. These findings reflect the findings of field research in high-risk populations within communities with a low prevalence of TB [12]–[15]. These studies evaluated the yield of source cases identified in close contact investigations of subjects from different age groups and populations, by comparisons with the yield of source cases from the investigations of close contacts of patients with pulmonary TB patient [12]–[15]. However, they were carried out in a country with a low incidence of TB and, to our knowledge, no study has compared the efficacy of these approaches with that of associated investigations based on TST levels in schoolchildren.

We show here that the investigation of close contacts of first-year schoolchildren with highly positive TST results increases the number of pulmonary TB cases detected by a factor of 1.6. A TST response of more than 5 mm in diameter was significantly associated with reported prior contact with a sputum smear-positive pulmonary TB patient. In children in their first year at school, BCG vaccination has been shown to have little effect on TST response in Madagascar [5]. This time point would therefore undoubtedly be the most appropriate for screening programs based on TST results.

In this study, we screened all the close contacts of the 90 schoolchildren for tuberculosis. The overall prevalence of active TB in this study population was therefore 2068 per 100,000 inhabitants for all forms of TB and 919 per 100,000 inhabitants for sputum smear-positive pulmonary TB. These figare five and eight times higher, respectively than the those in the NTP records (415/100,000 inhabitants and 111/100,000 inhabitants). Although Madagascar was thought to have reached the 70% case detection rate target, this analysis revealed a major lack of TB screening and control in Antananarivo. The higher prevalence observed in this study may be indicative of a high prevalence among the close contacts of schoolchildren with a TST response ≥5 mm in diameter. The detection of active cases in high-prevalence countries is not currently recommended by the WHO, but our results suggest that active case detection increases the number of cases treated and may, therefore, decrease the burden of tuberculosis, in accordance with the principal objectives of the WHO global anti-TB program.

Although the target population for this study were the family contact, due to the the high prevalence of TB level in Madagascar, the infection of some schoolchildren may have resulted from contact with individuals with pulmonary TB outside the household. TB infection may be acquired in the community due to close social contact at school, potentially bringing children into contact with TB cases when they visit friends or family or are themselves visited. Such persons would not be identified as current cases by this close contact investigation. This is consistent with the findings of Verver and Classen that, in an area with a high incidence of TB, the principal source of infection may be contacts outside the household [16], [17]. Even though these studies have been carried out with adult tuberculosis positive patients, it is conceivable that some positive TST results in these first year schoolchildren probably reflect not only TB transmission from close contacts, but also contact with TB cases in the community.

The interpretation of the TST may be confounded by prior vaccination with bacillus Calmette-Guérin (BCG), exposure to mycobacteria other than *Mycobacterium tuberculosis* and host immune status (Paediatric Tuberculosis Collaborative Group, 2004). However, we limited errors in the interpretation of TST response in this study by including only children with a BCG scar. Furthermore, Raharimanga *et al*. have shown that, in Antananarivo, a positive TST in schoolchildren is more likely to reflect TB infection than prior BCG vaccination [5]. Routine BCG vaccination at birth has frequently been reported to have only a minimal effect on the tuberculin response obtained in tests carried out several years later [18].

The schoolchildren and their close contacts in our survey were not tested for HIV infection. However, HIV infection rates in the general population of Madagascar have been estimated at only 0.14% (MoH, 2007), and it therefore seems likely that few, if any, of the children included in this study were infected.

We may have omitted some possible sputum smear-positive pulmonary TB cases among the close contacts using microscopic examination for diagnosis. Indeed, Culture remains the gold standard for the diagnosis of Tuberculosis. However, in many low-resource high-burden countries like in Madagascar, microscopic examination of Ziehl-Neelsen stained sputum specimens is often the only tuberculosis test available due to its low cost [19]. This introduce the possibility of a classification bias and underestimating the prior contact of the children with pulmonary TB in close contacts. Nevertheless, this is unlikely to have biased the study, because it would have affected all three TST groups and the subjects did not know their TST status when the information was requested.

## Conclusion

In conclusion, screening for active cases among the close contacts of children in their first year at school is a potentially applicable strategy, even in high-risk areas. Failure to control the spread of tuberculosis is largely due to the inability to detect and treat all infectious cases of pulmonary tuberculosis in a timely fashion allowing continued *Mycobacterium tuberculosis* transmission within communities. Tools for TB detection must be developed or improved in developing countries, which are the most strongly affected in terms of the number of cases and deaths from TB. Therefore, we suggest increased TST availability and access in developing countries.
